# Immunotherapy in NSCLC Patients With Brain and Leptomeningeal Metastases

**DOI:** 10.3389/fonc.2022.787080

**Published:** 2022-04-12

**Authors:** Thomas Pierret, Niccolò Giaj-Levra, Anne-Claire Toffart, Filippo Alongi, Denis Moro-Sibilot, Elisa Gobbini

**Affiliations:** ^1^ Thoracic Oncology Unit, Grenoble University Hospital, La Tronche, France; ^2^ Department of Advanced Radiation Oncology, Istituto di Ricovero e Cura a Carattere Scientifico (IRCCS) Sacro Cuore Don Calabria Hospital, Negrar di Valpolicella, Italy; ^3^ University of Brescia, Brescia, Italy; ^4^ Cancer Research Center Lyon, Center Léon Bérard, Lyon, France

**Keywords:** lung cancer, immunotherapy, brain, leptomeningeal, metastases

## Abstract

Immunotherapy has now been integrated as a treatment strategy for most patients with non-small cell lung cancer (NSCLC). However, the pivotal clinical trials that demonstrated its impressive efficacy often did not include patients with active, untreated brain metastases or leptomeningeal carcinomatosis. Nevertheless, NSCLC is the most common tumor to metastasize to the brain, and patients develop brain and meningeal involvement in approximately 40 and 10% of cases, respectively. Consequently, the appropriate care of these patients is a recurrent clinical concern. Although there are many aspects that would merit further investigation to explain the mechanism of intracranial response to immune checkpoint inhibitors (ICPs), some data suggest that they are able to cross the blood–brain barrier, resulting in local tumor microenvironment modification. This results in a similar clinical benefit in patients with stable, previously treated brain metastases compared to the general population. Despite important limitations, some real-life studies have described that the ICPs’ efficacy was maintained also in less selected patients with untreated or symptomatic brain metastases. In contrast, few data are available about patients with leptomeningeal carcinomatosis. Nevertheless, neurological complications due to ICP treatment in patients with brain metastases have to be evaluated and carefully monitored. Despite the fact that limited data are available in the literature, the purpose of this review is to show that the multimodal treatment of these patients with brain metastases and/or leptomeningeal disease should be discussed during tracing of the history of the disease, participating in the local and possibly systemic control of NSCLC.

## 1 Introduction

Non-small cell lung cancer (NSCLC) frequently metastasizes to the central nervous system (CNS) (1). Twenty percent of patients have brain metastases (BM) at the time of diagnosis, while in 40 and 10% of cases, they develop brain and leptomeningeal involvement, respectively, during tracing of the history of the disease (2–4). Due to the remarkable results achieved by immunotherapy and the improvement in our ability to detect and treat other sites of the disease, the number of patients with CNS metastases is expected to increase. Consequently, the appropriate care of these patients is a recurrent clinical concern.

Brain is traditionally considered an immune-privileged site, but some studies suggested that it can become accessible to immune check point inhibitors (ICPs) due to the blood–brain barrier disruption by brain tumors (5). Moreover ICPs, not attacking cancer cells itself, can remove the T-cell blockage, peripherally allowing functional T-cells to reach the brain and leptomeningeal metastasis (6, 7). However, the molecular profile and the tumor microenvironment of BM substantially differs from primary lung cancer, suggesting a potentially different effect by immunotherapy. Indeed genomic studies by next-generation sequencing on matched primary lung tumor and BM showed a significant heterogeneity in terms of somatic mutation and copy number alteration, potentially resulting in a different tumor mutational burden (8–10). Furthermore, lower T-cell density and PD-L1 expression besides a contracted T-cell receptor repertoire were found in BM compared to the matched lung primary tumor (10, 11). These observations suggested an immunosuppressive tumor microenvironment within the CNS niche that can potentially affect the immunotherapy efficacy regardless of the molecular and immunohistochemical features of the primary disease.

Although patients with CNS metastases generally have a poorer prognosis than the general population, this subgroup of patients is rather heterogeneous. Factors such as the number of metastatic lesions, their size, location, and associated symptoms as well as the control of extracranial disease may describe very different conditions in terms of prognosis (12). For these reasons, results obtained with ICPs in patients enrolled in clinical trials with treated, asymptomatic BM should not be generalized to the whole population with CNS involvement. Even less is known about the efficacy of this type of drugs in patients with leptomeningeal metastasis (LM).

Here we review the efficacy and safety of immune checkpoint inhibitors in NSCLC patients with brain and LM and the multimodality strategies that can be proposed to better control symptoms and local progression.

## 2 Immune Checkpoint Inhibitors Are Safe in NSCLC Patients With Metastasis to the Central Nervous System

### 2.1 Pre-Treated and Stable BM

Most studies exploring the efficacy of ICPs in NSCLC allowed the inclusion of patients with BM if the investigators provided that the intracranial disease was controlled and the patients were asymptomatic. Around 10% of patients included in clinical trials had controlled BM at diagnosis, providing interesting information about this group of patients as reported in a dedicated *post-hoc* analysis.

**Figure 1:**
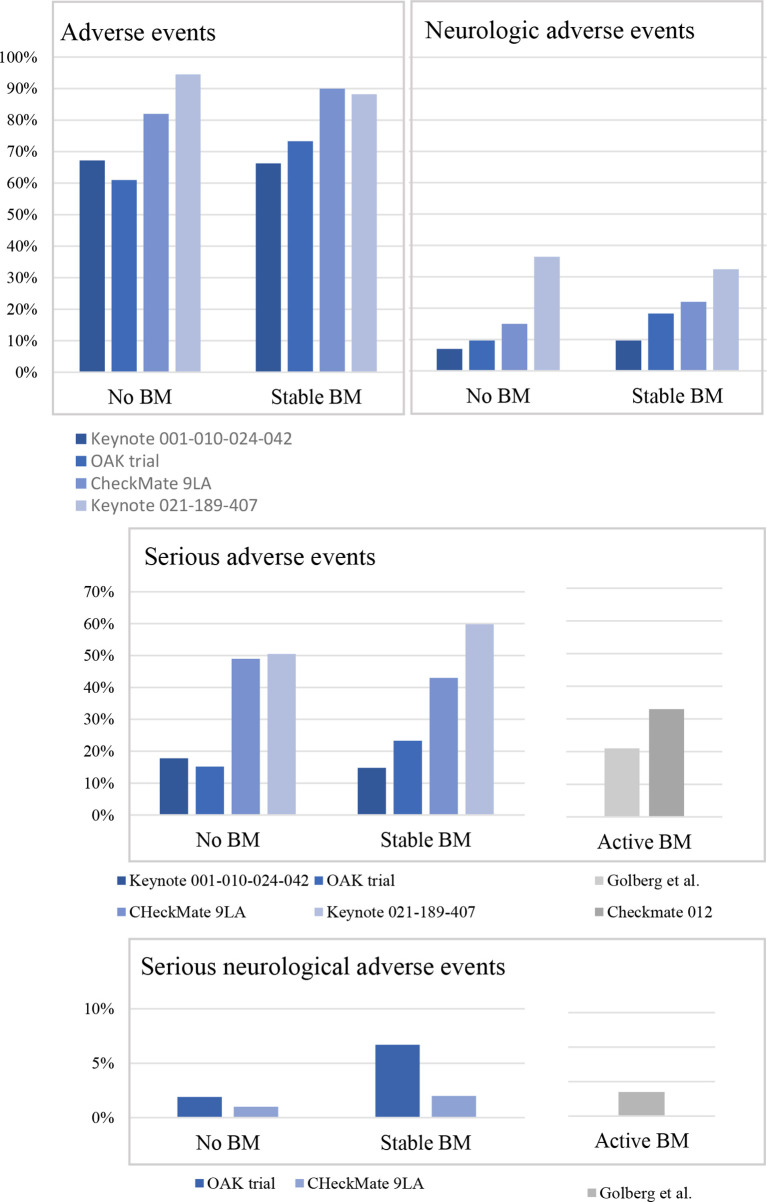
Safety in non-small cell lung cancer patients treated with immunotherapy according to the intracranial involvement. BM, brain metastases; Keynote 001-010-024-024, pembrolizumab arm only; Keynote 021-189-407, pembrolizumab plus chemotherapy only; OAK trial, atezolizumab only; CheckMate 9LA, nivolumab plus ipilimumab plus chemotherapy arm only.

An exploratory analysis of the phase III OAK trial evaluated the efficacy and safety of atezolizumab monotherapy *versus* docetaxel in pre-treated metastatic NSCLC patients with BM (13). The trial allowed the inclusion of patients with stable, pre-treated, and asymptomatic BM, thereby enrolling 123 patients (14%) with these characteristics. Grade ≥III treatment-related adverse events were observed in 23.3 and 15.2% of patients with and without BM who were receiving atezolizumab (**Figure 1**). Among them, severe neurologic adverse events appeared in 6.7% of patients with BM and in 1.9% of patients without BM who were receiving pembrolizumab. In particular, headache and dizziness were the most common symptoms.

According to this study, treatment-related neurological adverse events seem to be higher in patients with BM treated with atezolizumab compared to patients without BM. However, two recent pooled analyses provided completely different results.

In the pooled analysis from Keynote 001, Keynote 010, Keynote 024, and Keynote 42 trials, Mansfield et al. evaluated the efficacy and safety of pembrolizumab monotherapy *versus* chemotherapy in metastatic NSCLC patients with BM at baseline (14). Only patients with PD-L1 ≥1% were included in this *post-hoc* analysis. As previously published, these trials allowed BM only if previously treated and stable and if the patients did not need corticosteroids for symptoms (293 patients, 9.2% of the study population). According to the pooled analysis a similar safety profile was found between patients with and without BM who were receiving pembrolizumab (**Figure 1**). Despite the lack of information regarding the severity of symptoms, headache and dysgeusia were the most common neurologic treatment-related adverse events reported.

Similarly, the pooled analysis of Keynote 021, Keynote 189, and Keynote 407 trials reported the efficacy and safety of pembrolizumab combined with platinum-based chemotherapy *versus* chemotherapy alone in metastatic NSCLC patients with BM at baseline (15). In these trials, patients with pre-treated or untreated BM (Keynote 189 and Keynote 407 only) could be included, but the metastases had to be stable, and the patients did not need corticosteroids for symptoms (171 patients, 13% of the study population). Here again no differences were reported in terms of treatment-related adverse events in patients with BM compared to patients without BM treated with chemo-immunotherapy (**Figure 1**). Dysgeusia and peripheral neuropathy were the most common neurologic adverse events reported.

This results were supported by a recent subgroup analysis of Checkmate-9LA trial focusing on patients with BM at baseline (*N* = 101) (16). The patients included in this study received an association of platinum-based chemotherapy, nivolumab and ipilimumab or chemotherapy alone as upfront treatment. Once again, no safety signals were observed in patients with BM compared to patients without BM at baseline (**Figure 1**). Among neurological adverse events, dysgeusia and peripheral neuropathy were the most commonly reported.

Overall, according to these studies, safety profile of immunotherapy alone or combined with chemotherapy was similar in patients with and without baseline BM. Moreover, the presence of BM was not associated with an increased incidence of CNS adverse events in either treatment group.

### 2.2 Active Brain Metastasis

Active BM can be defined as newly diagnosed untreated lesions or growing lesions after a previous local treatment. Patients presenting these features are usually excluded from clinical trials, and only few studies reported information about the safety of immunotherapy in this subgroup of patients.

Teixeira Loiola de Alencar et al. reviewed all articles by focusing on this issue in the “lung cancer field” and exploring the efficacy and toxicity of immune checkpoint inhibitors (17). They selected 12 studies, most of which were retrospectives, but only 5 of them reported information about toxicity (18–22). The pooled results for grades 3 and 4 toxicity rate in patients with untreated BM was 20.5%. The 2 prospective studies included in this analysis provided interesting details.

In the phase II trial of Goldberg *et al.*, patients with 1 to 10 BM never pre-treated on progressing after a local treatment were enrolled to receive pembrolizumab until progression or up to 24 months (18). The study enrolled 42 patients, most of which were receiving pembrolizumab as first or second line of treatment. The most frequent neurological adverse events were headache (36% of cases), dizziness (24% of cases), cognitive disfunction (19% of cases), paraesthesia (14% of cases), and peripheral neuropathy (7% of cases). They were mostly grades 1 and 2, with the exception of grade 3 cognitive dysfunction, seizure, and stroke in one patient each, all judged unrelated to pembrolizumab (**Figure 1**). However, the attribution of these adverse events to pembrolizumab was complicated due to the fact that the study was not randomized.

The second study focused on by this issue is the multi-arm CheckMate 012 trial including patients with untreated, asymptomatic BM and no evidence of cerebral oedema (arm M) (19). Patients could be included if they had less than 4 BM of less than 3 cm each and they did not receive any local treatment before. However, patients had to have performed at least one line of systemic treatment. Twelve patients were enrolled and received nivolumab monotherapy up to disease progression. Four of them (33%) experienced at least one serious adverse event, and three patients (9.7%) discontinued the treatment because of toxicity (**Figure 1**). Only one patient experienced a neurological serious adverse event with seizure. Overall, neurological adverse events were rare (two patients experienced dizziness, two peripheral sensor neuropathy, one hypoesthesia, and one insomnia).

### 2.3 Leptomeningeal Metastasis

Limited data is currently available about the efficacy and safety of immune checkpoint inhibitors in patients with LM, with these latter usually excluded from clinical trials. Moreover, the few information we have was derived from pan-histology trials.

In a phase 2 trial, 20 patients in any line of treatment with advanced solid tumors with LM were treated with pembrolizumab (23). The patients had breast carcinoma (85%; *n* = 17), NSCLC (5%; *n* = 1), SCLC (5%, *n* = 1), and ovarian carcinoma (5%; *n* = 1). Nineteen of 20 patients (95%) had one or more adverse events that were considered to be at least possibly related to treatment, and eight patients (40%) had one or more grade 3 or higher adverse events. The neurologic adverse events included headache, dizziness, somnolence, and syncope, but they were of grade ≥ 3 (headache, *n* = 3; somnolence, *n* = 1; and syncope, *n* = 1).

In another phase 2 trial, 13 patients in any line of treatment with advanced solid tumors with LM were treated with single-agent pembrolizumab for 2 cycles (24). The patients who derived benefit from pembrolizumab could continue the treatment until disease progression or unacceptable toxicity. To be eligible for enrolment, the patients must not have an escalating steroid requirement. The patients had breast carcinoma (38.4%, *n* = 5), high-grade glioma (23.1%, *n* = 3), NSCLC (23%, *n* = 3), squamous cell carcinoma of the head and neck (7.6%, *n* = 1), and cutaneous squamous carcinoma of the skin (7.6%, *n* = 1). Five patients (38.5%) developed treatment-related adverse events, the most common being pain in the extremity, joint reduced range of motion, fatigue, and pruritus (*n* = 2 each). Immune-related adverse events were experienced by 3 patients (23.1%), none of which were of high grade in nature: pain in the extremity (*n* = 2), joint reduced range of motion (*n* = 2), pruritus (*n* = 2), maculopapular rash (*n* = 1), limb edema (*n* = 1), and pneumonitis (*n* = 1).

These results do not allow us to conclude about the toxicity of ICPs in patients with LM, but they suggest a toxicity profile similar to the general population.

## 3 Immune Checkpoint Inhibitors Are Effective in NSCLC Patients With Metastasis to the Central Nervous System

The activity of immunotherapy in patients with brain localizations has been recently confirmed despite the fact that most clinical trials did not allow patients with CNS metastases at baseline because of the risk of hyper-progression and difficulties in controlling brain edema without corticosteroids.

### 3.1 Pre-Treated and Stable Brain Metastases

#### 3.1.1 Single-Agent Immunotherapy

**Figure 2:**
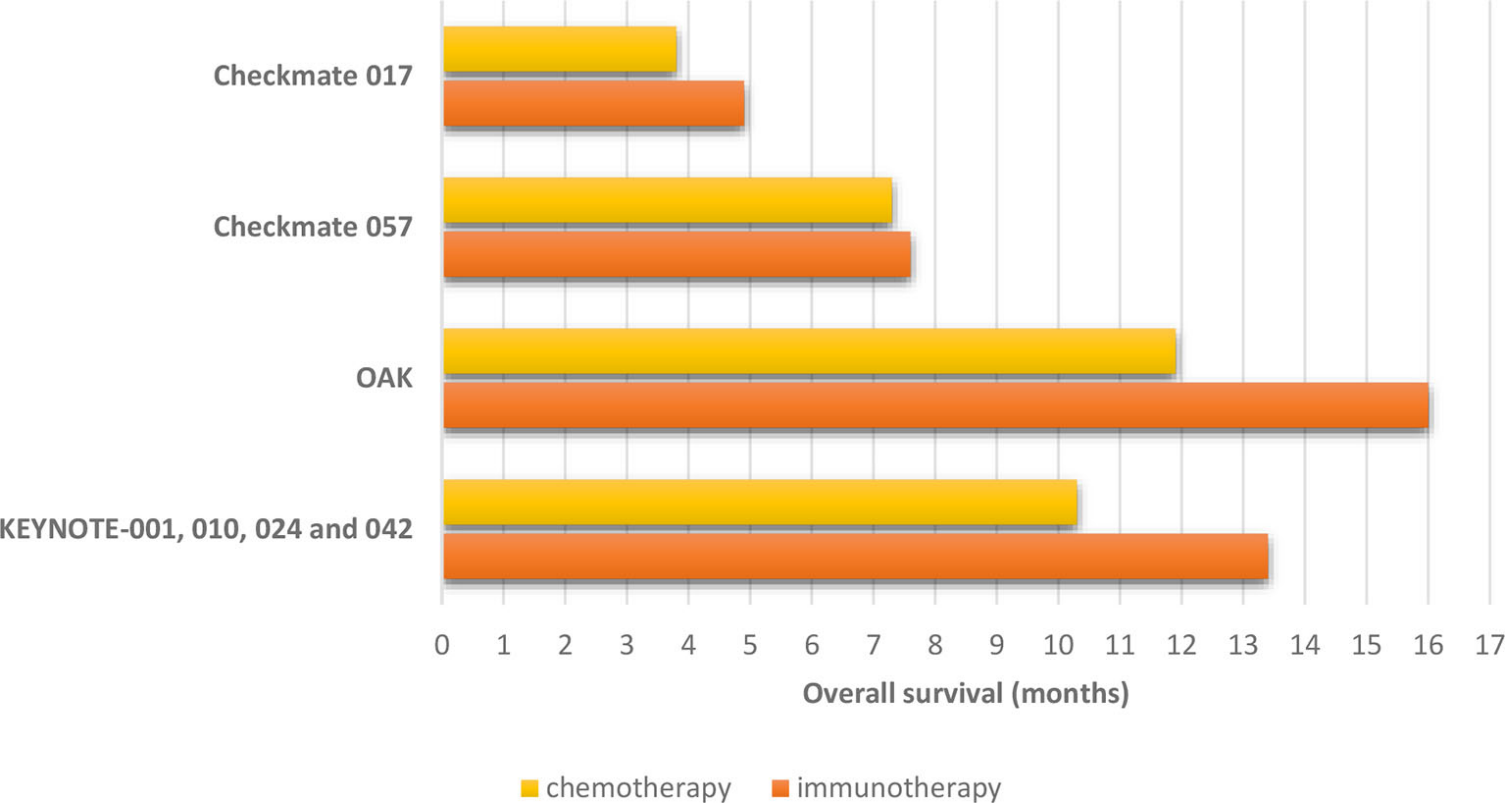
Overall survival in non-small cell lung cancer patients with pre-treated and stable brain metastases treated in randomized trials comparing immune checkpoint inhibitors to chemotherapy.

In the pooled analysis from Keynote 001, Keynote 010, Keynote 024, and Keynote 42 trials, the patients had received pembrolizumab or standard chemotherapy in different settings, including as first-line treatment (14). After a median follow-up of 12.9 months, pembrolizumab provided a longer overall survival compared to chemotherapy regardless of the presence of BM at baseline (HR 0.83 and HR 0.78 in patients with and without BM, respectively) (**Figure 2**). The magnitude of this benefit was even more important in patients PD-L1 ≥50% (HR 0.67 and HR 0.66, respectively). Similarly, the progression-free survival (PFS) was higher in the pembrolizumab group regardless of the brain metastatic status (HR 0.96 and HR 0.91 in patients with and without BM, respectively), with better results in patients with PD-L1 ≥50% (HR 0.70 and HR 0.69 in patients with and without BM, respectively).

Moreover, the pooled analysis of Checkmate-063, Checkmate-017, and Checkmate-057 trials was focused on a subgroup of patients with pretreated and stable BM at baseline (25). As previously published, in these trials, metastatic NSCLC patients received nivolumab after the failure of at least one line of treatment, and in Checkmate-017 and Checkmate-057, this strategy was compared to docetaxel. Globally, 46 patients were included in the pooled analysis, and most of them (74%) underwent a prior brain radiotherapy. Nivolumab was confirmed to provide OS benefit compared to docetaxel regardless of the brain metastatic status in both studies (**Figure 2**). At the time of disease progression or last tumor assessment, 33% of patients had no evidence of CNS progression (stable/decreased CNS lesions).

Finally, in the OAK study, comparing atezolizumab to docetaxel in patients that received at least one prior line of systemic treatment, atezolizumab was confirmed to provide survival benefit compared to docetaxel regardless of the presence of brain metastasis (13) (**Figure 2**). Furthermore, the risk of developing new BM appeared to be lower in the atezolizumab arm compared to docetaxel (median time to new CNS lesion development: not reached *versus* 9.5 months, HR: 0.42, 95% CI: 0.15–1.18) in patients with baseline BM.

#### 3.1.2 Association of Immunotherapies

**Figure 3:**
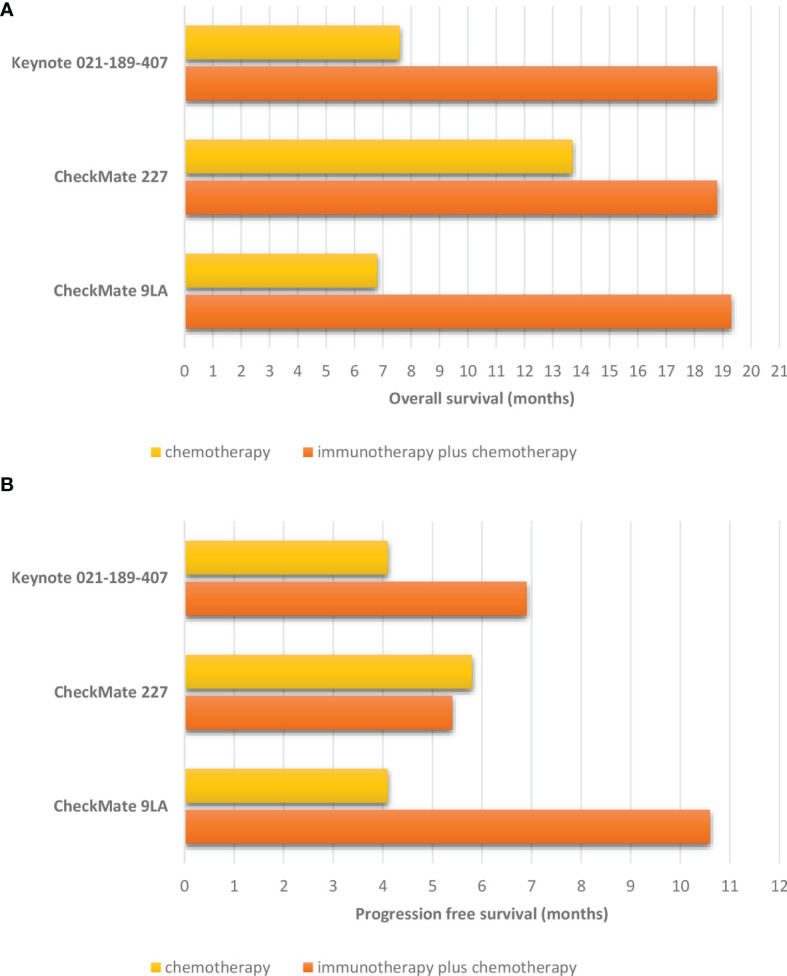
Survival in non-small cell lung cancer patients with pre-treated and stable brain metastases treated in randomized trials comparing immune checkpoint inhibitors combined with chemotherapy to chemotherapy alone. **(A)** Overall survival. **(B)** Progression free survival.

The Checkmate-227 trial enrolled treatment-naïve metastatic NSCLC patients to receive a standard platinum-based chemotherapy, a combination of nivolumab and ipilimumab, or nivolumab as a single agent if PD-L1 ≥1% or associated with chemotherapy if PD-L1 negative (26). The study allowed patients with treatment of asymptomatic BM, and a *post-hoc* analysis was focused on this subgroup (27). Here again, patients that received the double immunotherapy got a significant clinical benefit in OS (HR, 0.57) and PFS (HR, 0.79) despite the presence of BM (**Figure 3**). The duration of responses was longer with the association of nivolumab and ipilimumab than with chemotherapy (24.9 and 8.4 months, respectively).

#### 3.1.3 Immunotherapy Combined With Chemotherapy

The pooled analysis of Keynote-021, Keynote-189, and Keynote-407 trials investigated the outcomes of NSCLC patients with BM treated with pembrolizumab combined with a platinum-based chemotherapy or with chemotherapy alone (15). The overall response rate (ORR) in patients with BM in the pembrolizumab plus chemotherapy arm was 39% compared to 54.6% in patients without BM. Moreover, patients with BM treated in the pembrolizumab arm achieved an improved OS (HR, 0.48) and PFS (HR, 0.44) as in the general population. Finally, the duration of response was longer with pembrolizumab plus chemotherapy *versus* chemotherapy alone regardless of BM and PD-L1 status.

Similarly, a recent subgroup analysis of Checkmate-9LA trial focusing on patients with BM at baseline has been reported. The patients included in this study received an association of 2 cycles of platinum-based chemotherapy, nivolumab and ipilimumab, or chemotherapy alone as upfront treatment. In a subgroup of patients treated by immunotherapy and chemotherapy combination, 86% have been treated by radiotherapy before the beginning of treatment. After a minimum follow-up of 23.3 months, a better ORR was found in the combination arm compared to the chemotherapy arm (43 *versus* 24%). Moreover, longer PFS (HR, 0.40) and OS (HR, 0.43) were recorded in the group of patients treated with chemo-immunotherapy compared to chemotherapy alone (**Figure 3**). The median intracranial PFS in the association arm was interesting being 13.5 months (4.6 months in the chemotherapy arm; HR, 0.36). Finally, the median time to development of new brain lesions was longer with chemo-immunotherapy (9 *versus* 4.6 months) (16).

### 3.2 Active Brain Metastases

Several retrospective studies have suggested the immunotherapy efficacy in patients with active BM.

A European multicenter study included 1,025 patients treated by anti-PD-1 or anti-PD-L1 monotherapy in the first line (28). In this cohort, 255 patients (24.9%) had BM (39.2% active, 14.3% symptomatic, and 27.4% receiving steroids). The ORR in patients with BM *versus* those without BM was similar (20.6 *versus* 22.7%, respectively), while the median PFS was 1.7 and 2.1 months, respectively (*p* = 0.009). The median OS was 8.6 months in patients with BM and 11.4 months in those without BM (*p* = 0.035).

Moreover, in the phase II study of Goldberg et al., patients with NSCLC or melanoma with untreated BM and no neurological symptoms or corticosteroid requirement received pembrolizumab (18) (**Table 1**). After a median follow-up of 8.3 months (4.5–26.2), 11 out of 37 patients had a CNS response (29.7%; 95% CI, 15.9–47), and the CNS progression-free survival was 2.3 months (95% CI, 1.9-NR).

**Table 1:** Ongoing clinical trials with immune checkpoint inhibitors in NSCLC patients with untreated brain metastases.

Study (phase)	Population	Inclusion criteria	Experimental arm	Primary endpoint
NCT02681549 (II)	NSCLC and melanoma	At least one untreated BM 5–20 mm, asymptomatic, steroids off, PD-L1 positive	Pembrolizumab + bevacizumab	Intracranial response rate
NCT02886585 (II)	NSCLC and melanoma	Untreated asymptomatic BM or progressive ≥10 mm or cytology-positive neoplastic meningitis	Pembrolizumab	ORR, extracranial ORR, overall survival
NCT03526900 (II)	NSCLC	Untreated BM, asymptomatic, and ≤4 mg dexamethasone/day	Atezolizumab + carboplatine + pemetrexed followed by pemetrexed and atezolizumab	PFS

NSCLC, non-small cell lung cancer; BM, brain metastases; ORR, objective response rate; PFS, progression-free survival.

Finally, the Checkmate-012 trial (arm M) included 12 patients with up to 4 asymptomatic and untreated BM to receive nivolumab (19). In this phase I trial, the patients had at least one prior systemic therapy, and their PD-L1 status was unknown. Two intracranial responses were observed (16.7%; 95% CI, 2.1–48.4). The median OS was 8.0 months (95% CI, 1.38–15.5), and the median PFS was 1.6 months (95% CI, 0.92–2.5). It is interesting to note that one of the responders had leptomeningeal involvement, too.

Recently, the phase II study Atezo-Brain explored the efficacy of atezolizumab associated with carboplatin and pemetrexed as first-line treatment in patients with untreated BM at baseline (29). The study included 40 patients with a median follow-up of 17.3 months. Seventy patients (43%) had baseline corticoids. The intracranial ORR was 40%, the median PFS was 8.9 months (95% CI, 6.7–13.8), and the median OS was 13.6 months. The median intracranial PFS was 6.9 months (95% CI, 4.7–12.1).

Further studies are needed to confirm that immunotherapy can be a treatment option for patients with active BM, but these results seem encouraging (**Table 1**).

### 3.3 Leptomeningeal Metastases

Literature about immunotherapy efficacy in patients with LM are too poor to draw any conclusion. Two phase II trials were dedicated to patients with leptomeningeal involvement from different solid tumors treated with pembrolizumab in advanced settings.

In the first one, Naidoo et al. included 3 NSCLC patients (24). The primary endpoint was the CNS response (radiological, cytological, or clinical) after four cycles. Thirteen patients were enrolled, and a CNS response at 12 weeks was observed in 38% of cases (95% CI, 13.9–68.4%). The median CNS PFS and OS were 2.9 months (95% CI, 3.7–NR) and 4.9 months (95% CI, 3.7–NR), respectively. One patient with NSCLC achieved complete response.

The other phase II trial included only one NSCLC patient (23). The study met its primary end-point, which was the proportion of patients alive at 3 months (HR, 0.60; 90% CI, 0.39–0.78). Twenty patients were included, and they showed a median OS of 3.6 months (90% CI, 2.2–5.2). Subsequent studies are needed to explore immunotherapy activity in patients with leptomeningeal metastases (**Table 2**).

**Table 2:** Ongoing trials with immune checkpoint inhibitors in patients with leptomeningeal metastasis.

Study (phase)	Population	Phase	Inclusion criteria	Primary endpoint
NCT04356222	NSCLC	IV	Durvalumab + methotrexate IT	OS, NPFS, AE
NCT04729348	All tumor	II	Pembrolizumab + lenvatinib	OS at 6 months
NCT02939300	All tumor	II	Nivolumab + ipilimumab	OS
NCT03719768	All tumor	I	Avelumab + WBRT	DLT

NSCLC, non-small cell lung cancer; IT, intrathecal; WBRT, whole-brain radiation; OS, overall survival; NPFS, neurologic progression-free survival; AE, adverse events; DLT, dose-limiting toxicity.

## 4 Local Treatment Can Improve Intracranial Control in Patients Treated With Immunotherapy

Radiation therapy is still recognized as an important oncological strategy in BM. In the recent years, the radiation oncology community is gradually promoting the use of stereotactic radiosurgery (SRS) or hypofractionated radiotherapy treatments (HypoRT), compared to whole brain radiotherapy (WBRT), when a limited number of BM are detected (30). In fact, WBRT is associated with a higher probability of neurocognitive deterioration (31). Additionally, a phase III randomized clinical trial demonstrated that WBRT did not improve the OS when compared to best supportive care in patients with BM and NSCLC (32). Consequently, the prescription of WBRT should be offered to selected patients with neurological symptoms but not eligible to local SRS or HypoRT treatments and with good performance status (30).

The combination of immunotherapy and radiotherapy is an intriguing approach supported by several pre-clinical studies, in which a synergistic antitumor activity has been demonstrated (33). The initial clinical experience is a phase I trial, KEYNOTE-001, demonstrating that a previous treatment with radiotherapy (intra- and extracranial), followed by pembrolizumab in 97 patients with advanced NSCLC, resulted in longer PFS and OS, with an acceptable safety profile (34).

### 4.1 WBRT and Immunotherapy

The current clinical experiences published about the combination of WBRT and immunotherapy in NSCLC are very limited.

The largest published retrospective study evaluated the toxicity profile in brain metastatic patients enrolled to receive a radiation treatment (WBRT, SRS, or partial brain irradiation) with or without immunotherapy (nivolumab, pembrolizumab, and atezolizumab) (35). A total of 163 patients were included, and the majority received WBRT (62%). The authors did not report any significant differences in terms of adverse events with the combined strategy compared to exclusive intracranial radiation treatment.

Focusing on exclusive WBRT in a retrospective analysis, Doi et al. explored the potential prognostic factors which should be taken into account in the combination of intracranial radiotherapy and systemic treatment (including immunotherapy). The authors reported a significant improvement in terms of OS in PD-L1-positive patients, supporting the potential role of combining immunotherapy and WBRT in this group (36). In a small series including 5 patients with NSCLC and BM, the safety impact of WBRT and immunotherapy has been evaluated. Apparently, the authors did not report a higher probability of neurocognitive toxicity when a combined approach was offered (37).

According to the limiting prescription of WBRT in metastatic NSCLC patients, definitive conclusions about the combination of immunotherapy and radiotherapy are not achievable.

### 4.2 SRS or HypoRT and Immunotherapy

Several experiences have been published about the combination of SRS or HypoRT and immunotherapy. Shepart et al. showed disappointing results when comparing, in a retrospective match analysis, the use of SRS and immunotherapy to SRS alone (38). Apparently, the combination of SRS and immunotherapy was correlated with a higher probability of intracranial progression. On the other side, the authors did not report a good tolerability in terms of side effects and radionecrosis risk.

Conversely, Shapira et al. evaluated in a retrospective series the impact in terms of OS and loco-regional control in 37 patients treated with SRS/HypoRT and immunotherapy (nivolumab, pembrolizumab, atezolizumab) (39). At a median follow-up of 14.3 months, OS probability was higher in patients who received a concomitant treatment of radiotherapy and immunotherapy compared to those who had radiotherapy performed after immunotherapy (OS at 1 year: 87.3 *versus* 0%). These results were confirmed also in terms of distant brain failure (defined as the appearance of new brain metastases) and local control. An excellent tolerability was recorded.

Similar positive results in terms of clinical outcomes and tolerability were reported in other retrospective series (40, 41). According to the majority of retrospective studies, a period between 4 weeks and 1 months before or after immunotherapy could be defined as the cutoff to differentiate a concurrent or sequential treatment approach (42).

Currently, different phase II clinical trials are ongoing to evaluate, in a prospective study, the impact of radiotherapy and concurrent immunotherapy (NCT04291092, NCT04787185). Another phase II trial is exploring the use of SRS followed by immunotherapy *versus* immunotherapy followed by radiotherapy (NCT04650490).

## 5 Conclusion

The ability of immunotherapy to cross the blood–brain barrier was already known, but its intracranial activity was uncertain. According to recent evidence, the immune checkpoint inhibitors seem able to keep their anticancer activity against active or treated CNS metastases despite an unfavorable environment. Indeed the pooled analyses of pivotal trials showed that immunotherapy is effective in improving the outcome for NSCLC patients regardless of the presence of BM. Moreover, there was no evidence of increased general or neurological toxicity in this subgroup of patients. Less is known about the role of immunotherapy in patients with LM. The few patients that received this strategy within dedicated clinical trials did not show a significant clinical benefit, given that their prognosis remained very poor. However, future studies with larger numbers of patients are needed, and some clinical trials are ongoing in this setting (**Tables 1**, **2**).

Moreover, local ablative treatment combined with immunotherapy seems to be effective and safe. Indeed some data suggest that the concurrent combination of immunotherapy and radiotherapy can be associated with a better outcome compared to immunotherapy alone in patients with BM. If the results of prospective ongoing trials will validate this positive signal, the standard of care of patients with BM at baseline would be the association of immunotherapy and intracranial radiation treatment.

## Author Contributions

EG, TP, and NG-L performed literature research and were in charge of manuscript writing. All authors contributed to the article and approved the submitted version.

## Conflict of Interest

The authors declare that the research was conducted in the absence of any commercial or financial relationships that could be construed as a potential conflict of interest.

## Publisher’s Note

All claims expressed in this article are solely those of the authors and do not necessarily represent those of their affiliated organizations, or those of the publisher, the editors and the reviewers. Any product that may be evaluated in this article, or claim that may be made by its manufacturer, is not guaranteed or endorsed by the publisher.
